# Role of Neuropilin 1 in COVID-19 Patients with Acute Ischemic Stroke

**DOI:** 10.3390/biomedicines10082032

**Published:** 2022-08-20

**Authors:** Asma W. Al-Thomali, Hayder M. Al-kuraishy, Ali I. Al-Gareeb, Ali K. Al-buhadiliy, Michel De Waard, Jean-Marc Sabatier, Atif Ali Khan Khalil, Hebatallah M. Saad, Gaber El-Saber Batiha

**Affiliations:** 1 Department of Biology, College of Science, Taif University, P.O. Box 11099, Taif 21944, Saudi Arabia; 2 Department of Pharmacology, Toxicology and Medicine, Medical Faculty, College of Medicine, Al-Mustansiriyah University, MBChB, MRCP, FRCP, Baghdad P.O. Box 14132, Iraq; 3 Department of Pharmacology, Toxicology and Medicine, Medical Faculty, College of Medicine, Al-Mustansiriyah University, Baghdad P.O. Box 14132, Iraq; 4 Department of Clinical Pharmacology, Medicine and Therapeutic, Medical Faculty, College of Medicine, Al-Mustansiriyah University, Baghdad P.O. Box 14132, Iraq; 5 Smartox Biotechnology, 6 rue des Platanes, 38120 Saint-Egrève, France; 6 L’institut du Thorax, INSERM, CNRS, UNIV NANTES, 44007 Nantes, France; 7 LabEx «Ion Channels, Science & Therapeutics», Université de Nice Sophia-Antipolis, 06560 Valbonne, France; 8 Institut de Neurophysiopathologie (INP), Aix-Marseille Université, CNRS UMR 7051, Faculté des Sciences Médicales et Paramédicales, 27 Bd Jean Moulin, 13005 Marseille, France; 9 Department of Biological Sciences, National University of Medical Sciences, Rawalpindi 46000, Pakistan; 10 Department of Pathology, Faculty of Veterinary Medicine, Matrouh University, Matrouh 51744, Egypt; 11 Department of Pharmacology and Therapeutics, Faculty of Veterinary Medicine, Damanhour University, Damanhour 22511, Egypt

**Keywords:** COVID-19, neuroinflammation, acute ischemic stroke, neuropilin-1

## Abstract

Severe acute respiratory syndrome coronavirus type 2 (SARS-CoV-2) infection can trigger the adaptive and innate immune responses, leading to uncontrolled inflammatory reactions and associated local and systematic tissue damage, along with thromboembolic disorders that may increase the risk of acute ischemic stroke (AIS) in COVID-19 patients. The neuropilin (NRP-1) which is a co-receptor for the vascular endothelial growth factor (VEGF), integrins, and plexins, is involved in the pathogenesis of AIS. NRP-1 is also regarded as a co-receptor for the entry of SARS-CoV-2 and facilitates its entry into the brain through the olfactory epithelium. NRP-1 is regarded as a cofactor for binding of SARS-CoV-2 with angiotensin-converting enzyme 2 (ACE2), since the absence of ACE2 reduces SARS-CoV-2 infectivity even in presence of NRP-1. Therefore, the aim of the present study was to clarify the potential role of NRP-1 in COVID-19 patients with AIS. SARS-CoV-2 may transmit to the brain through NRP-1 in the olfactory epithelium of the nasal cavity, leading to different neurological disorders, and therefore about 45% of COVID-19 patients had neurological manifestations. NRP-1 has the potential capability to attenuate neuroinflammation, blood–brain barrier (BBB) permeability, cerebral endothelial dysfunction (ED), and neuronal dysfunction that are uncommon in COVID-19 with neurological involvement, including AIS. Similarly, high NRP-1 serum level is linked with ED, oxidative stress, and the risk of pulmonary thrombosis in patients with severe COVID-19, suggesting a compensatory mechanism to overcome immuno-inflammatory disorders. In conclusion, NRP-1 has an important role in the pathogenesis of COVID-19 and AIS, and could be the potential biomarker linking the development of AIS in COVID-19. The present findings cannot provide a final conclusion, and thus in silico, experimental, in vitro, in vivo, preclinical, and clinical studies are recommended to confirm the potential role of NRP-1 in COVID-19, and to elucidate the pharmacological role of NRP-1 receptor agonists and antagonists in COVID-19.

## 1. Introduction

The present pandemic coronavirus disease 2019 (COVID-19), a recent respiratory disease pandemic, is caused by a severe acute respiratory syndrome coronavirus-2 (SARS-CoV-2) [1,2]. The majority of COVID-19 patients present with mild to moderate symptoms, though 15% of them may progress to acute COVID-19 pneumonia and acute lung injury (ALI). Moreover, 5% of COVID-19 patients may eventually progress to acute respiratory distress syndrome (ARDS) and multi-organ failure (MOF) with secondary severe septic shock [3,4]. COVID-19 is usually treated by symptomatic therapy with antiviral drugs like remdesivir. However, assisted mechanical ventilation is required for COVID-19 patients with ARDS and respiratory failure [5]. Despite the development of authorized COVID-19 vaccines, treatments that target immunopathology and immuno-thrombosis have become a great focus.

It has been documented that SARS-CoV-2 infection can trigger adaptive and innate immune responses leading to uncontrolled inflammatory reactions and associated local and systematic tissue damage [6]. In severe COVID-19 there is a momentous lymphopenia, eosinopenia, basopenia, monocytopenia, and a reduction in B and T cell numbers with marked neutrophilia and high neutrophil-lymphocyte ratio (NLR), which indicates disease severity [7].

On the other hand, COVID-19 is linked with coagulopathies that mimic other systemic coagulation disorders, which are connected with severe infections such as thrombotic microangiopathy and disseminated intravascular coagulation (DIC) [8]. Nonetheless, COVID-19 has a distinctive feature due to the development of endothelial dysfunction (ED), pro-hemostatic abnormality, and unrecognized thromboembolic complications. Notably, these coagulation disorders are associated with high D-dimer, prolongation of prothrombin time, and a significant reduction in platelet counts [8]. These findings suggest that COVID-19-induced coagulopathy is linked with a low grade of DIC and pulmonary micro-thrombosis.

**Figure 1:**
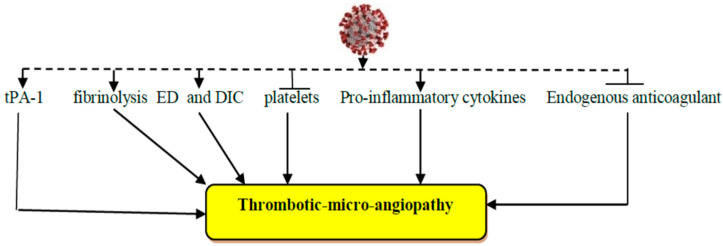
Thrombotic micro-angiopathy in COVID-19: SARS-CoV-2 infection induces the release of tissue-plasminogen activator (tPA), fibrinolysis, and the release of pro-inflammatory cytokines with the development of endothelial dysfunction (ED). In addition, SARS-CoV-2 infection decreases platelets and the release of endogenous anticoagulants.

Moreover, SARS-CoV-2 infection can induce the release of pro-inflammatory cytokines including interleukin (IL)-6, tumor necrosis factor-alpha (TNF-α), and IL-1β [9]. It has been proposed that pro-inflammatory cytokines and developed cytokine storm could inhibit the endogenous anticoagulant pathway [10,11]. Remarkably, SARS-CoV-2 infection may affect the fibrinolytic pathway, which is an important factor linked with mortality. In addition, tissue plasminogen activator is reported to be elevated in patients with severe COVID-19 [12,13]. Moreover, SARS-CoV-2 infection-induced ED could induce the release of plasminogen activators, which might explain a high D-dimer in COVID-19 patients [8]. Similarly, SARS-CoV-2 infection-induced thrombotic microangiopathy is typically related to the interaction between platelets and the von Willebrand factor, which is released from the injured endothelium [14]. Different experimental studies have revealed that the von Willebrand factor is disintegrated by a disintegrin and metalloprotease thrombospondin type 1 member 13 (ADAMTS13), which is reduced in severe systemic infections [15,16]. These findings suggest that SARS-CoV-2 infection is associated with substantial coagulation disorders (Figure 1).

Significantly, the incidence of thromboembolic complications in critically ill patients is 5–15%, while in COVID-19 this incidence is increased up to 35–45% due to augmented platelet activation, ED, thrombo-embolic profile, and a deficiency of anti-fibrinolytic activity [17,18]. These changes may increase the risk of acute ischemic stroke (AIS) in COVID-19 patients.

Furthermore, different clinical studies have highlighted the importance of inflammatory biomarkers in the diagnosis and prognosis of COVID-19 patients. For example, Hu et al. revealed that procalcitonin serum level was correlated with COVID-19 severity and mortality [19]. In addition, N-terminal pro-brain natriuretic peptide (NT-proBNP), troponin-T, lactate dehydrogenase (LDH), and creatin kinase (CK) levels reflect the severity of the acute cardiac injury, ARDS, and MOF [20,21,22].

**Figure 2:**
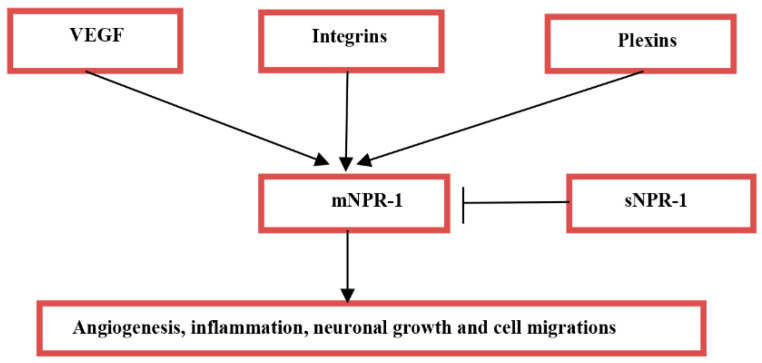
The legends of neuropilin-1 receptors (NRP-1): Vascular endothelial growth factor (VEGF), integrins, and plexins activate membrane-bound NRP-1 (mNPR-1) leading to angiogenesis, inflammation, and neuronal growth and cell migrations. Though, soluble NPR-1 (sNPR-1) inhibits action of mNPR-1.

Moreover, the molecular mechanism of neuronal injury in AIS is still a matter of debate and has been the subject of intense research and investigations. Different studies have shown that various transcription and tissue factors are involved in the pathogenesis of AIS [23]. One of the important molecules is neuropilin (NRP), which is a receptor for semaphoring polypeptide also called plexin. There are two types of NRPs, NRP1 and NRP2, which have short intracellular domains and trans-membrane protein (non-tyrosine kinase). NRP-1 has two isoforms, including secretory (as truncated or soluble NRP-1) and transmembrane isoforms. The former circulates freely in the body fluid, while the latter interacts with various ligands and has different functions [24]. They act as co-receptors for the vascular endothelial growth factor (VEGF), integrins, and plexins (Figure 2) [25]. In addition, NRP acts as a receptor for axon growth, and as a guidance factor, such as semaphoring P3A (SMAP3A). NRP overexpression is linked with vascular formation and neuronal guidance in cancer, and targeting of the NRP gene during embryonic development leads to insufficient vascularization and neuronal pattering [26]. NRP/SMAP3A, in addition to its protection against AIS, also has important pathological sequences in the pathogenesis of different neurodegenerative disorders [27].

Therefore, the aim of the present study was to elucidate the potential role of NRP-1 in COVID-19 patients with AIS.

## 2. Acute Ischemic Stroke and Neuropilin-1

AIS is defined as focal neurological deficits that persist for more than 24 h due to vascular ischemia and injury [28]. AIS often leads to irreversible neuronal damage and associated complications like focal seizure and permanent motor-sensory deficits [28]. AIS is more common after the age of thirty, although it can happen at any age, even in childhood. AIS represents the chief cause of long-term disability and accounts for 11.9% of annual deaths. AIS represents 90% of total strokes [29]. The most common causes of AIS are thromboembolic complications, atrial fibrillation, plaque instability in atherosclerosis, and microangiopathy/neuroinflammation; AIS without a known cause is called cryptogenic stroke [29]. The risk factors for AIS are old age, male gender, inherited factors, hypertension, diabetes mellitus, obesity, alcoholism, smoking, oral contraceptive pills, and cardio-metabolic disorders [30].

AIS is commonly associated with injury of the blood–brain barrier (BBB) due to reactive gliosis, neuro-inflammation, and oxidative stress with hemostatic dysfunction [31]. In AIS there is a significant interaction between neurons, glial cells, and matrix components that are collectively called a neurovascular unit [32]. Neuro-inflammation and peri-infarct immunological activations lead to more neuronal injury and anterograde and retrograde axonal degenerations [33]. Moreover, AIS leads to excitotoxicity and neuronal oxidative stress resulting in exaggerated ischemia, abnormal neuronal glucose metabolism, and neuronal calcium overload in the infracted and peri-infarcted regions [34]. At this moment different inflammatory and pro-inflammatory cytokines are released causing vasculitis and BBB injury due to the activation of microglial cells and infiltrating macrophages in the infracted area. A high level of inflammatory/pro-inflammatory cytokines initiates aggressive neuroinflammation with the induction of apoptosis [35].

On the other hand, the over-expression of NRP-1 and VEGF is associated with increased vascular angiogenesis and density during AIS of experimental models and humans [36]. Zhang et al. showed that the administration of NRP-1 or VEGF may enhance AIS and improve neurological recovery through the induction of angiogenesis [37]. NRP-1 is highly expressed in the neurons, astrocytes, and endothelial cells that play an important role in the regulation of functional processes following AIS. Thus, NRP-1 is upregulated after experimental AIS in rats [38]. Likewise, the overexpression of NRP-1 in transgenic mice causes excessive neovascularization, while mice with NRP-1 knockout revealed insufficient neovascularization and neuroprotection [39]. Therefore, NRP-1 is vital for vascular development and neuroprotection, since neovascularization is essential to preventing deterioration after focal AIS [40].

**Figure 3:**
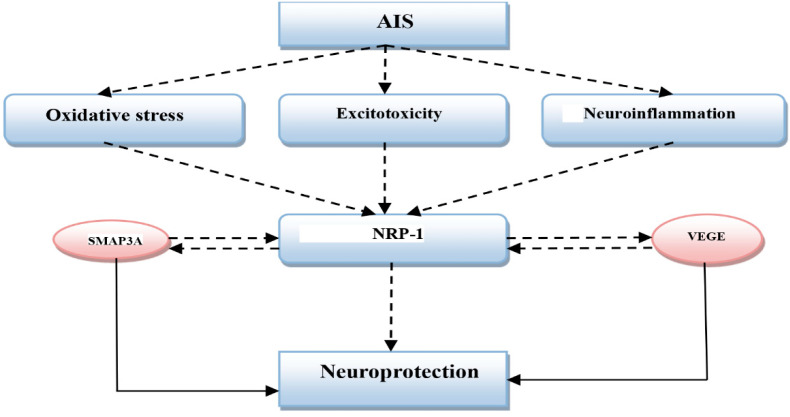
Role of neuropilin-1 receptors (NRP-1) in acute ischemic stroke (AIS): AIS triggers the development of oxidative stress, excitotoxicity, and neuroinflammation that activate the expression of NRP-1. In turn, NRP-1 activates expressions of vascular endothelial growth factor (VEGF) and semaphoring P3A (SMAP3A), which stimulate NRP-1 receptors leading to neuroprotection.

In addition, NRP-1 increases the expression of VEGF, which is also upregulated during AIS for induction of neovascularization. Moreover, the persistent elevation of NRP-1 following the angiogenesis period suggests that NRP-1 plays an essential role in the remodeling and maturation of new blood vessels after AIS [41]. Adults with AIS revert to the prenatal pattern of NRP-1 expression with higher expression in the cerebral cortex leading to the augmentation of the effects of VEGF and SMAP3A [42]. Therefore, NRP-1, which is a potential receptor for both VEGF and SMAP3A, has a protective role against AIS. These findings suggest that high NRP-1 in AIS is regarded as a compensatory mechanism against the deteriorative neuronal effects of neuroinflammation during AIS (Figure 3).

In contrast, ample studies have observed that AIS and other cerebral ischemia in humans and animals are linked with neuronal modeling and neovascularization. An experimental study by Zhang et al. showed that NRP-1 overexpression in concert with VEGF is linked to neovascularization in adult rats with AIS [38]. Moreover, Jiang and colleagues demonstrated that NRP-1 mediates the axonal damage induced by the E2 promoter binding factor 1 (E2F1) transcription factor. Therefore, NRP-1 inhibitors might be neuroprotective against neuronal excitotoxicity and deprivation of glucose oxygen metabolisms during AIS [43].

Human studies have indicated that post-stroke cellular events are involved in neuronal regeneration through the NRP-1-dependent pathway [43]. Mehta et al. revealed that endothelial NRP-1/VEGF signaling is activated by disintegrin and metalloproteinases (ADAMs). So, ADAM inhibitors may reduce NRP-1 activity and NRP-1-induced VEGF activation [44]. Clinical studies have shown that mild AIS induces the release of VEGF, which promotes angiogenesis and protects ischemic neurons from damage, encouraging neuronal plasticity through anti-inflammatory effects. However, severe AIS prompts BBB injury and may exacerbate brain injury [45]. Slevin et al. disclosed that VEGF serum level is correlated with the severity of AIS [46]. These findings suggest that the immunological reaction to AIS is mediated by VEGF, which could be of great value in assessing the status and recovery of patients with AIS.

Therefore, VEGF through an NRP-1 or NRP-1 independent pathway may be involved in the pathogenesis of AIS. Thus, the modulation of the VEGF/NRP-1 axis could be of great value in the management of AIS.

## 3. Acute Ischemic Stroke in Viral Infections

AIS is not uncommon with various types of viral infections, since acute and chronic viral infections can provoke AIS through alterations of the underlying atherosclerotic process and immune-hematological reactions [47]. Indeed, AIS has an undesirable impact on the immune response against viral infections due to AIS-induced immunodepression causing profound post-stroke complications. So, post-AIS infection is regarded as an independent predictor of poor clinical outcomes [47]. Manousakis et al. divulged that AIS disrupts the protective mechanisms against acute viral infections via the induction of immunosuppression and release of anti-inflammatory cytokines. Therefore, the risk of viral infections following AIS is augmented via a paramount immunological circuit [48].

During respiratory viral infections (RTIs), the platelet activation and reactivity are augmented with an increase in the pro-aggregatory state, which increases the risk of AIS [49]. Different case-control studies have confirmed the close relationship between RTIs and AIS [50]. It has been shown that the influenza viral infection is associated with an increase in the risk of AIS in the elderly population that is reduced following influenza vaccination [51].

A prospective study involving 170 patients with RTIs compared with 80 healthy controls revealed that platelet activity is increased and linked with the severity of RTIs [49]. Both P-selectin and platelet CD62 are amplified, indicating platelet overactivity with ED, alteration of lipid metabolism, and plaque stability [49]. P-selectin and activated platelets are augmented during RTIs, with subsequent risk of AIS due to platelet–monocyte interactions and the release of pro-inflammatory cytokines [52]. Moreover, circulating activated platelets during RTIs exacerbate plaque instability and rupture with succeeding thromboembolic complications mainly in patients with cardio-metabolic disorders [53].

An in vivo study by Ferroni et al. showed that hepatitis C virus (HCV) infection is associated with platelet activation and the risk of AIS. Moreover, total cholesterol, factor VII, high-density lipoprotein, and platelet count were lower in patients with HCV compared to healthy controls, though total P-selectin was higher compared with controls and correlated with viral load [54]. Moreover, human immune deficiency virus (HIV)-infected patients experienced platelet activation and high P-selectin serum level [55]. In addition, platelets from HIV-infected patients exhibited an activated apoptotic pathway and mitochondrial dysfunction [55]. Therefore, the prevalence of HCV infection is higher in AIS patients compared with controls due to the high inflammatory burden and fibrinogen level [55]. Therefore, acute and chronic viral infections are associated with an increase in the risk of AIS due to low-grade inflammatory reactions and platelet overactivity.

On the other hand, NRP-1, which is linked with the development of AIS, is also associated with different viral infections. Ghez et al. showed that NRP-1 is regarded as a receptor and an entry point for human T-cell lymphotropic virus type 1 (HTLV-1) [56]. NRP-1 is highly expressed in dendritic cells, T cells, and endothelial cells, which are the chief target of HTLV-1 infection. Interestingly, T cell activation during HTLV-1 infection is connected with the upregulation of NRP-1 receptors [56]. Moreover, NRP-1 promotes the entry and infectivity of the Epstein–Barr virus (EBV) via direct interaction of EBV glycoprotein B fusion protein with the NRP-1 receptor [57]. Recently, Wang et al. demonstrated that the NRP-1 decoy receptor can neutralize the viral infectivity of enterovirus A71 (EVA71) due to the interaction between the soluble extracellular domain of NRP-1 with the viral proteins [58]. Likewise, NRP-1 is regarded as a receptor for the entry of cytomegalovirus (CMV), and is required for viral gene expression, replication, and induction of necroptosis [59]. Therefore, the elimination of NRP-1 receptors by specific antagonists or the use of soluble NRP-1, which neutralizes viral particles, could reduce CMV infectivity [59].

**Figure 4:**
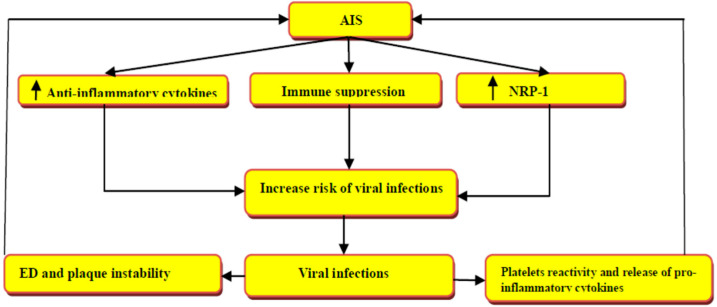
Viral infections and the risk of acute ischemic stroke (AIS): AIS induces the expression of neuropilin-1 receptors (NRP-1) and the release of anti-inflammatory cytokines with the development of immunosuppression. Acute viral infections lead to endothelial dysfunction (ED), plaque instability, platelets hyper-reactivity, and the release of pro-inflammatory cytokines—changes that increase the risk of AIS.

These findings indicate that NRP-1 is crucial for the entry and pathogenesis of various types of viruses, and the blocking of this receptor may attenuate the pathogenesis of different viral infections. Since NRP-1 receptors are highly expressed in the neurons, NRP-1 receptor antagonists not only reduce viral infectivity but also the risk of associated AIS mainly in susceptible patients (Figure 4).

## 4. Immunological Role of Neuropilin-1

NRP-1 has important immunological effects in preventing and limiting autoimmunity and immunopathology through maintaining and preserving immune homeostasis. NRP-1 has momentous antiviral and anti-tumor effects through the regulation of the immunity/inflammatory axis [60]. NRP-1 improves and regulates regulatory T- cells (Treg) function by directing the expression of the transcription factor (Foxp3), which has a foremost role in the programming and development of Treg [61]. The interactions of NRP-1 with Treg limit the development of experimental colitis through the enhancement of survival and quiescence with the suppression program cell death of Treg [62]. In addition, NRP-1 inhibits autoreactivity in experimental autoimmune encephalitis (EAE) through the regulation of immune response and Treg function against myelin constituents. Moroever, CD4+ T cells expressing NRP-1 receptors inhibit the proliferation and pro-inflammatory cytokine production from effectors T cells independent of Treg cells [63].

Tordjman et al. showed that the initiation of primary immune response needs contact between resting T cells and dendritic cells (DCs) [64]. This interaction is mainly mediated by the expression of NRP-1 receptors on both T cells and DCs, leading to the augmentation of NRP-1 receptors on T cells. Thus, the blockade of NRP-1 receptors inhibits DC-mediated T cells activation [64]. These verdicts suggest that NRP-1 receptors are essential for the interaction between T cells and DCs during the primary immune response, and NRP-1 could be the link between the immune system and neuronal inflammation. In addition, NRP-1 positively regulates the immune response to cancer cells and malignant tumors through the regulation of CD8+ infiltration into solid tumors [65]. NRP-1 is regarded as a potential marker for CD4+/FoxP3 Treg cells, which regulate immune response against malignant cells [65]. However, the interaction of the NRP-1 receptor with its semaP3A ligand limits the migration of tumor-specific cytotoxic T cells [65]. Thereby, in vivo immunotherapeutic inhibition of NRP-1 receptors enhances tumor control, cytotoxicity, and the proliferation of CD8+ T cells. Likewise, the blockade of NRP-1 receptors, which act as an immune memory checkpoint, may trigger the development of tumor-specific memory T cells having anti-tumor immunity [66].

Notably, TLR4 is essential for tolerance against HSP-70, which is a self-antigen. Lipopolysaccharide (LPS) stimulates the expression of TLR4 while HSP-70 inhibits the expression of TLR4 to regulate cytokine release and innate immune response [67]. NRP-1 inhibits the expression of TLR4 by regulating the inflammatory signaling pathway in a model of sepsis [68]. Moreover, NRP-1 improves the expression of HSP-70 in rats with experimental AIS [69]. Therefore, NRP-1 may modulate the immunological response during AIS by regulating the expression and activity of the TLR4/HSP-70 axis.

Concerning the immunological response and role of NRP-1 during neuroinflammation, it has been observed that NRP-1 knockdown increases brain microvascular endothelial cells inflammation through the suppression of anti-inflammatory cytokines with the activation of signal transducer activator transporter 1 (STAT1) [70]. NRP-1 preserves BBB, inhibits neuronal demyelination, and lymphocyte infiltration with suppression of inflammatory signaling in the neurons [70]. Therefore, NRP-1 plays a potential role in the prevention of brain microvascular endothelial dysfunction in various forms of neuroinflammation [70]. Further, secondary neuroinflammation following the development of AIS promotes additional neuronal injury and brain cell death, though this neuroinflammation could be beneficial in enhancing the recovery from AIS [71].

Normally, NRP-1 expression is very low in normal neurons; nevertheless, it is augmented after AIS and associated neuroinflammation, and exerts beneficial effects in recovery from AIS via the modulation function of Treg immune response [71]. Thus, the deletion of NRP-1 enhances CD4+ T-cell-mediated disease severity due to attenuation of immunosuppressive effects of Treg cells during AIS [72]. Experimental studies have demonstrated that CD4+ T cells expressing NRP-1 receptors are extremely resistant to disease development and progression through a transforming growth factor beta (TGF-β)-dependent pathway [73]. Thus, NRP-1 acts as a central signaling pathway that regulates immune response and neuroinflammatory disorders.

**Figure 5:**
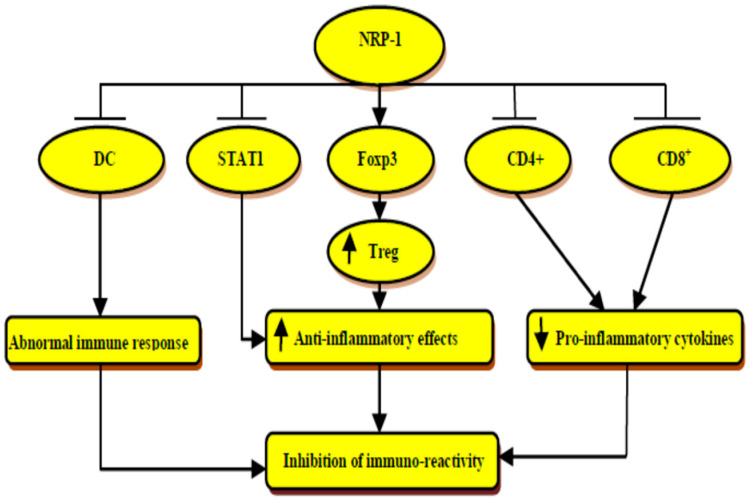
The immunological role of neuropilin-1 (NRP-1): Through transcription factor (Foxp3) and inhibition of signal transducer activator transporter 1(STAT1) NRP-1 activates regulatory T cell (Treg) and the release of anti-inflammatory cytokines, respectively. NRP-1 inhibits the release of pro-inflammatory cytokines through the inhibition of CD4+ and CD8+. NRP-1 through the SUPPRESSION of dendritic cells (DCs) attenuates abnormal immune response. These changes that are mediated by NRP-1 inhibit the development of immunological over-activity.

Moreover, the chronic inflammatory process negatively regulates and controls NRP-1 to induce Treg cells instability with the activation of TH17 cells. Thus, chronic inflammatory disorders trigger the reprogramming of Treg cells toward TH17 cells via the NRP-1-dependent pathway [74]. Taken together, the high NRP-1 serum level could be a compensatory mechanism to repress inflammatory and immunological instabilities (Figure 5).

## 5. Role of Neuropilin-1 in COVID-19

**Figure 6:**
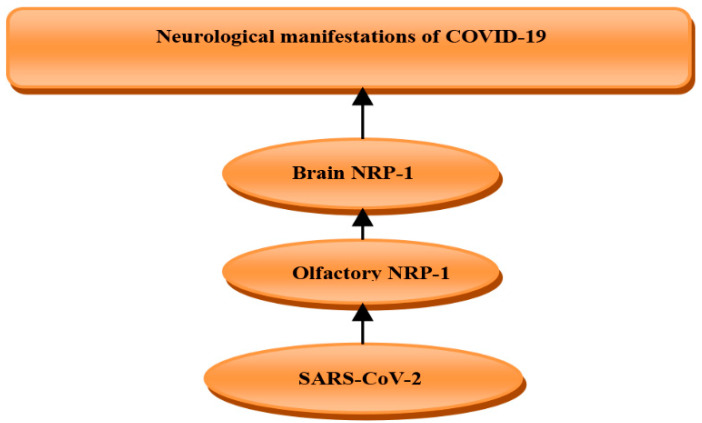
The role of neuropilin-1 (NRP-1) in SARS-CoV-2 infection: SARS-CoV-2 binds olfactory NRP-1 and is transmitted to the brain leading to induction of neurological manifestations of COVID-19.

It has been confirmed by different studies that NRP-1 is a co-receptor for the entry of SARS-CoV-2 and facilitates its entry to the brain through the olfactory epithelium. Therefore, SARS-CoV-2 through NRP-1 in the olfactory epithelium of the nasal cavity may transmit to the brain leading to different neurological disorders including headache, confusion, hallucination, and convulsion [75]. It has been confirmed that about 45% of COVID-19 patients had neurological manifestations [75]. Thus, NRP-1 inhibitors might be a promising therapeutic that may reduce SARS-CoV-2 infection-induced neurological complications [75]. SARS-CoV-2 spike protein contains two subunits S1 (for receptor binding) and S2 (for fusion and replication). Remarkably, NRP-1 precipitates S1 and binds more strongly to the S2 than S1 [76]. Moreover, the depletion of NRP-1 reduces the uptake and binding of SARS-CoV-2 to the host cells, so tissues with higher expression of NRP-1 are at risk for SARS-CoV-2 infection [77]. NRP-1 is regarded as a cofactor for the binding of SARS-CoV-2 with angiotensin-converting enzyme 2 (ACE2), since the absence of ACE2 reduces SARS-CoV-2 infectivity even in presence of NRP-1 [78]. This finding suggests that NRP-1 facilitates the communication and binding between SARS-CoV-2 and ACE2. In silico study has demonstrated that the receptor binding domain (RBD) of SARS-CoV-2 interacts preferentially with the b1 domain of NRP-1 [79], (Figure 6).

Furthermore, NRP-1 expression is higher in specific brain regions including the hippocampus, basal ganglion, thalamus, hypothalamus, brainstem, cerebral cortex, amygdala, and retina. Explicitly, the hippocampal formation consistently expressed the highest levels of NRP-1 genes and proteins. Therefore, NRP-1 may serve as a superfluous SARS-CoV-2 infection mediator involved in the neurologic manifestations of COVID-19 [80].

Moreover, the mRNA of NRP-1 is mainly expressed in brain macrophages, endothelial cells, oligodendrocytes, and astrocytes [81]. On the other hand, the expression of olfactory NRP-1 and other receptors for SARS-CoV-2 entry is low in newborns [82], which might explain mild neurological and other COVID-19 symptoms in newborns.

In COVID-19, the pathological consequences are results from direct SARS-CoV-2 cytopathic effects and exaggerated immune response [5]. It has been known that Treg cells maintain immunological homeostasis and prevent immunological disturbances in viral and autoimmune diseases. Treg cells inhibit different immune cells such as CD4+, CD8+, natural killer cells, monocytes, and B cells that are involved in autoimmunity [83]. Treg cells exert inhibitory effects by the secretion of IL-10 and TGF-β that interfere with the maturation and proliferation of CD4+ and CD8+ T cells [83]. Moreover, FoxP3 improves the development and functioning of Treg cells. FOXP3 is also upregulated in all effector T cells and it is not limited to Treg cells [84]. FOXO1 and other transcription factors may delineate the fate of a T cell. [84]. Normally, Treg cells are of two types: either natural Treg cells derived from thymus, or induced during the development of immunological tolerance [85]. In severe SARS-CoV-2 infection, Treg cells are inhibited, leading to an exaggerated immune response and the development of a cytokine storm [86]. A cytokine storm is defined as a systemic inflammatory response, which can occur as a result of a variety of factors, including infections and certain drugs, leading to the excessive release of pro-inflammatory cytokines [87]. It has been demonstrated that NRP-1 upregulates Treg cells [61], so may reduce exaggerated immune response-induced COVID-19 severity.

**Figure 7:**
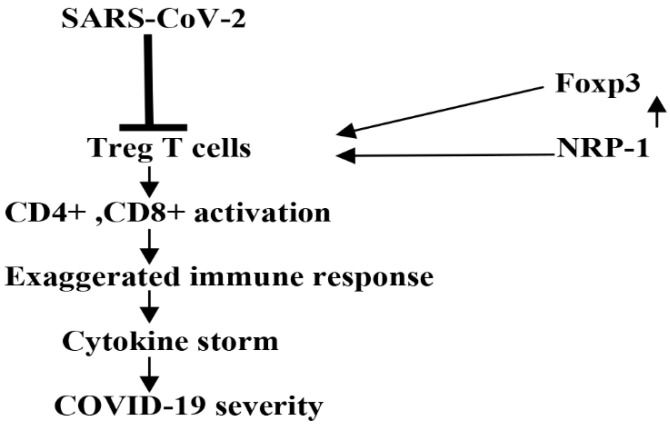
The role of neuropilin-1 (NRP-1) in SARS-CoV-2 infection-induced abnormal immune response: SARS-CoV-2 infection inhibits regulatory T cells (Treg), causing abnormal activation of CD4+ and CD8+, and the development of a cytokine storm.

Interestingly, FoxP3, which improves the expansion of Treg cells’ function is severely downregulated in SARS-CoV-2 infection and coincides with COVID-19 severity [88]. Mohebbi et al. study revealed that FoxP3 might be a potential biomarker for the assessment of COVID-19 severity [89]. Moreover, NRP-1 advances the expression of the FoxP3/Treg cells axis [90]. Therefore, NRP-1/FoxP3 is an important axis that regulates Treg cells’ function, prevents exaggerated immune response in COVID-19, and could be a possible prognostic factor for patients with severe COVID-19 (Figure 7).

Indeed, NRP-1 has an important function in maintaining the integrity of BBB during neuroinflammation and AIS. It has been shown that the neuronal inflammatory process induces the expression of miR-24, which increases the expression of NRP-1 mRNA in brain endothelial cells. High miR-24 also reduces BBB permeability in response to high circulating VEGF, a potent ligand of NRP-1 receptors [91]. Gambardella et al. clarified that miR-24 has prognostic and diagnostic roles in different diseases including COVID-19 [92]. Correspondingly, miR-24 target and modulate NRP-1 receptors in brain endothelial cells and are linked with cerebrovascular events including AIS in COVID-19.

In a cohort study involving 58 COVID-19 patients, miR-24 serum level was reduced compared to non-COVID-19 patients [92]. It has been confirmed that miR-24 serum level could be reduced secondary to high NRP-1 in COVID-19 [93]. A case-controlled study by Zhou et al. based on 68 patients with AIS compared with 21 healthy controls revealed that the miR-24 serum level was reduced in patients with AIS [94]. These findings indicate that a reduction of miR-24 serum level in COVID-19 patients heralds the development of AIS.

**Figure 8:**
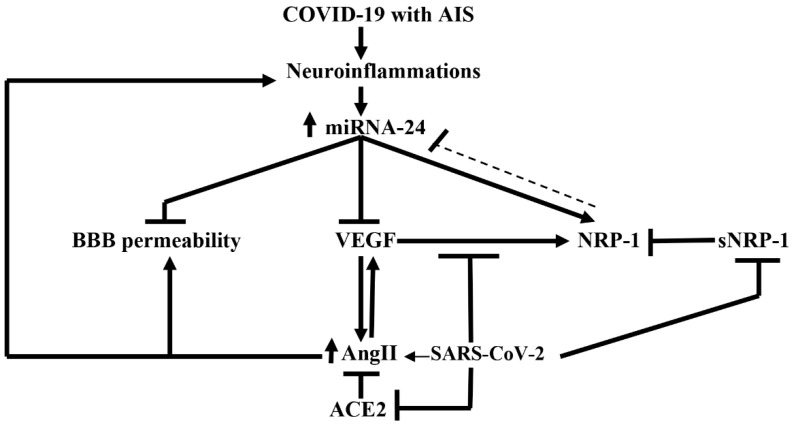
Role of miRNA-24 in COVID-19 with acute ischemic stroke (AIS): COVID-19 with AIS induces neuroinflammation and the release of miRNA-24, which inhibits blood–brain barrier permeability and the release of vascular endothelial growth factor (VEGF) with induction expression of NRP-1. The interaction between VEGF and NRP-1 is inhibited by SARS-CoV-2 leading to high NRP-1, which suppresses miRNA-24. SARS-CoV-2 inhibits soluble NRP-1(sNRP-1), an antagonist of NRP-1; moreover, SARS-CoV-2 increases angiotensin II (AngII), which induces neuroinflammation and increases the permeability of BBB. SARS-CoV-2 downregulates angiotensin-converting enzyme 2 (ACE2), which metabolizes AngII.

Furthermore, VEGF, which is an agonist of the NRP-1 receptor, is elevated in COVID-19 due to the blocking of VEGF/NRP-1 signaling by the spike protein of SARS-CoV-2, with subsequent development of neuropathic pain [95]. It has been indicated that soluble NRP-1 is regarded as a potent NRP-1 receptor antagonist that may increase the level of VEGF [96]. Moreover, VEGF/NRP-1 signaling dysfunction is associated with anosmia and other olfactory dysfunctions in SARS-CoV-2 infection. NRP-1 expression is highly linked to olfactory dysfunction and COVID-19 severity due to the involvement of CNS [97]. Yin et al. confirmed that VEGF is highly concerned and linked with neuroinflammation in COVID-19 [98]. VEGF is extensively distributed in the brain and plays a vital role in the development and progression of neuroinflammation by facilitating and recruiting inflammatory cells, and controlling the action of brain angiotensin II (AngII) [98]. Therefore, VEGF antagonists could be a promising target in the management of COVID-19-induced neurological manifestations. Recently, Chen et al. observed that VEGF has an important role in the progression of AIS, and plays a crucial role in the development of brain edema, collateral damage, atherosclerosis, and early pathological disorders in stroke patients [99]. Thus, high circulating VEGF serum levels in COVID-19 indicate neurological complications, including AIS. Moreover, an experimental study demonstrated that AngII induces the expression and production of VEGF mRNA, which is abolished by AngII receptor 1 (AT1) blockers like losartan [100]. Zhao et al. showed that AngII-induced inflammation is mainly mediated by the induction expression of VEGF in the vascular endothelium [101]. Al-kuraishy et al. disclosed that SARS-CoV-2 infection is linked with the downregulation of ACE2, which metabolizes AngII to the vasodilator Ang1-7 [102]. It has been stated that ACE2 inhibits the expression of VEGF mRNA [103], and thus the downregulation of ACE2 with high AngII triggers the release of VEGF in COVID-19, with the risk of the development of AIS (Figure 8).

**Figure 9:**
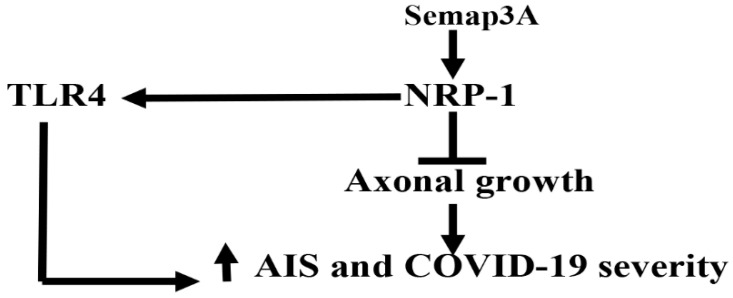
Role of semaphoring P3A (SemaP3A) in COVID-19 with acute ischemic stroke (AIS): Through activation of NRP-1, SemaP3A inhibit axonal outgrowth and expression of toll-like receptor 4 (TLR4), causing the augmentation of COVID-19 severity in AIS.

Notably, semaP3A, which acts on the NRP-1 receptor, may inhibit axonal outgrowth in experimental model studies. Therefore, semaP3A inhibitors could accelerate the functional recovery after AIS through the suppression of astrocyte activation in the peri-infarct area [104]. Nedaei et al. showed that lentiviral-induced expression of soluble NRP-1 inhibits semaP3A-mediated collapse activity in multiple sclerosis [26]. Soluble NRP-1 is protective against neuronal inflammation by attenuating the semaP3A/NRP-1 signaling pathway [26]. Furthermore, recovery from AIS is limited by the inhibitory environment in the post-ischemic brain region through the semaP3A-eicosanoid pathway, so the blocking of this pathway may improve recovery after AIS [105]. Moreover, the semaP3A/NRP-1 pathway is required for the activation of toll-like receptor 4 (TLR4), which plays an integral role in the development of immunological disturbances during COVID-19 [106]. These findings suggest that the semaP3A/NRP-1 pathway is involved in the progression of harmful consequences in both AIS and COVID-19 (Figure 9).

In COVID-19, pulmonary thrombosis and acute vascular distress syndrome are correlated with poor clinical outcomes and mortality [107]. Moreover, NRP-1 increases the expression and release of tissue factors from endothelial cells leading to the induction of thrombogenic events with the consumption of fibrinogen [108]. Therefore, a high NRP-1 level indicates the underlying development of pulmonary micro-thrombosis in patients with severe COVID-19. Significantly, pulmonary micro-thrombosis could be associated with AIS and other venous-thrombotic complications due to ED-mediated thromboembolic disorders [8,109]. These findings suggest that AIS and/or pulmonary thrombosis might be the presenting features of COVID-19.

**Figure 10:**
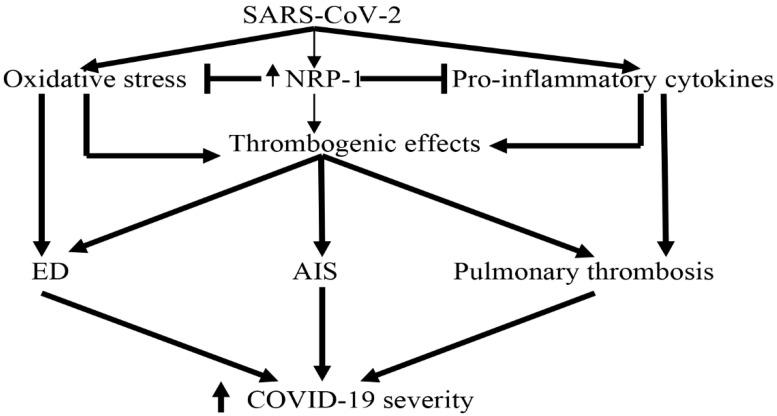
SARS-CoV-2 infection and thrombogenic effects: SARS-CoV-2 infection induces oxidative stress and the release of pro-inflammatory cytokines, and increases NRP-1 level that leads to thrombogenic effects, which lead to endothelial dysfunction (ED), acute ischemic stroke (AIS), and pulmonary thrombosis.

On the other hand, oxidative stress during SARS-CoV-2 is linked with the development of ED, pulmonary thrombosis, and MOF due to the generation of reactive oxygen species (ROS) and the reduction of endogenous antioxidant capacity [110]. In turn, high oxidative stress increases the oxidation residue of ACE2, with subsequent augmentation of its binding with the spike protein of SARS-CoV-2 leading to progressive hyperinflammation and serious complications [110]. Various studies have confirmed that NRP-1 has an antioxidant effect by improving mitochondrial function through inhibition generation of ROS and iron accumulation in the inner mitochondrial membrane [111]. In addition, antioxidants promote the mitochondrial function in NRP-1-deficient endothelial cells [111]. Thus, a high NRP-1 serum level in COVID-19 could be a compensatory mechanism to overcome oxidative stress injury and mitochondrial dysfunction (Figure 10).

Furthermore, various metabolic disorders are associated with the reduction of soluble NRP-1(sNRP-1), which has protective effects against AngII-induced inflammation [112,113]. In addition, sNRP-1 is up-regulated in HIV-positive pregnant women due to mimicry with VEGF or integrin, which mediate angiogenesis via NRP-1 receptors [114]. Interestingly, polycystic ovary syndrome (PCOS) when linked with metabolic syndrome is associated with the low level of sNRP-1 due to high circulating AngII, which inhibits the function of sNRP-1 and increases COVID-19 severity [114]. Therefore, high AngII in COVID-19 might be the possible cause of low sNRP-1 with high membrane-bound NRP-1 activity (mNRP-1).

**Figure 11:**
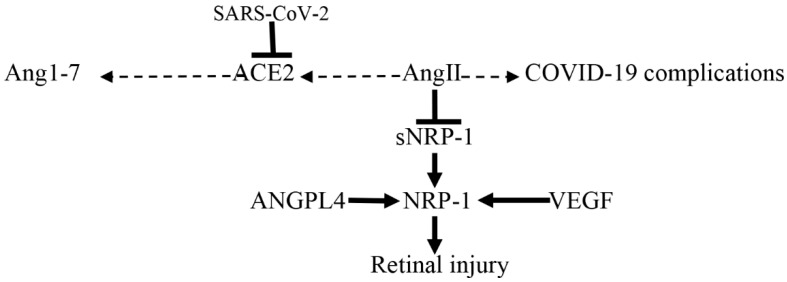
Role of neuropilin 1(NRP-1) in retinal injury in COVID-19: SARS-CoV-2 infection downregulates angiotensin-converting enzyme 2 (ACE2), increases angiotensin II (AngII), which inhibits soluble NRP-1(sNRP-1), leading to increased expression of NRP-1. In retinal tissue, vascular endothelial growth factor (VEGF) and angiopoietin-like peptide 4 (ANGPTL4) activate retinal NRP-1 receptors leading to retinal injury.

In addition, the high NRP-1 serum level in COVID-19 reflects retinal injury due to the over-expression of NRP-1 receptors in retinal ganglion cells that provokes retinal micro-hemorrhages [77]. Naidoo et al. showed that angiopoietin-like peptide 4 (ANGPTL4) and VEGF bind retinal endothelial cells NRP-1 receptors, causing retinal vascular leakage and the development of diabetic retinopathy [114]. These findings suggest that sNRP-1 and mNRP-1 play important roles in the regulation of inflammatory and oxidative pathways in COVID-19, mainly in patients with underlying metabolic and retinal disorders (Figure 11).

ANGPTL4 is a multi-functional protein implicated in different pathological disorders including cardio-pulmonary diseases, atherosclerosis, diabetes mellitus, malignancies, and nephritic syndrome [115]. ANGPTL4 is hydrolyzed by membrane convertase into N-terminal (nANGPTL4) and C-terminal parts (cANGPTL4). The nANGPTL4 inhibits lipoprotein lipase, the enzyme involved in the hydrolysis of circulating triglyceride under exercise and fasting conditions [116]. Thus, the inhibition of nANGPTL4 could be a therapeutic target in the treatment of hyper-triglyceridemia and increased insulin sensitivity [117]. ANGPTL4 activates both NRP-1 and NRP-2 in endothelial cells leading to the augmentation of endothelial permeability. Therefore, the administration of sNRP-1 may antagonize ANGPTL4 by blocking NRP-1 receptors [118].

It has been reported that ANGPTL4 was upregulated in lung tissue samples from patients during influenza pandemics in 2009. Influenza infection triggers the expression of ANGPTL4 through signal transducer and activator transcription 3 (STAT3)-dependent mechanisms, leading to the exacerbation of lung inflammation and injury. Therefore, anti-ANGPTL4 neutralizing antibodies may improve tissue integrity and recovery from influenza and pneumococcal pneumonia [119,120]. In addition, the high ANGPTL4 level is associated with the development of ALI and acute coronary syndrome [121,122]. High ANGPTL4 is positively correlated with the risk of atherosclerosis and ischemic stroke [123]. Moreover, an experimental study showed that high ANGPTL4 in AIS plays an important role in the reduction of infarct size and amelioration of neurological deficits by antagonizing the upregulated VEGF effects [124]. Therefore, high ANGPTL4 in AIS could be protective against the neurological sequels through the regulation permeability of endothelial cells and BBB.

**Figure 12:**
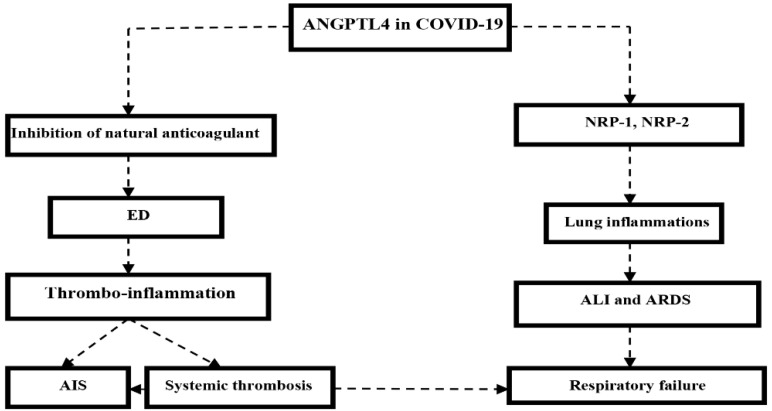
Role of angiopoietin-like peptide 4 (ANGPTL4) in COVID-19: ANGPTL4 through activation of NRP-1 and NRP-2 receptors induces lung inflammation, acute lung injury (ALI), and acute respiratory distress syndrome (ARDS) with the development of respiratory failure. ANGPTL4 through inhibition of natural anticoagulants causes endothelial dysfunction (ED) and the development of thrombo-inflammation leading to systemic thrombosis and acute ischemic stroke (AIS).

In COVID-19, a cohort study involving 64 COVID-19 patients compared with 50 patients with community-acquired pneumonia, showed that ANGPTL is increased in COVID-19 patients and correlated with disease severity compared to the controls [125]. Several studies have shown that a high ANGPTL level is correlated with high CRP and D-dimer in COVID-19 patients, and is regarded as a good predictor for admission to the intensive care unit [126,127,128]. ANGPTL may induce vasculitis and vasculopathy through the inhibition of thrombomodulin-mediated anticoagulant proteins and protein C [127]. These verdicts indicate that ANGPTL, a biomarker of ED, is elevated in COVID-19 due to the development of ED and associated thrombo-inflammation, which may cause the development of AIS. Thus, ANGPTL—mainly ANGPTL4 through the NRP-1 receptor—could be the potential link between COVID-19 and AIS (Figure 12).

Moreover, NRP-1 interacts and cooperates with platelet-derived growth factor (PDGF) and TGF-β to control various physiological and pathological responses including liver cirrhosis and fibrosis [128]. NRP-1 suppresses the anti-inflammatory and antithrombotic pathways through interaction with hypoxia-inducible factor-alpha 2 (HIF-α2), which inhibits platelet aggregation and macrophage activation [129]. It has been reported that PDGF together with pro-inflammatory cytokines are linked with COVID-19 severity [129]. Moreover, PDGF is elevated following stroke and correlates with the risk of vasospasm. Released PDGF binds receptor tyrosin kinase with the activation of MAPK, which induces vascular smooth muscle contractility and proliferation [130]. Recently, Zhang et al. study showed that TGF-β is linked with the development of AIS severity, and a high TGF-β serum level can be used as an early diagnostic and prognostic biomarker [131]. In addition, SARS-CoV-2 infection may induce the release of inactive TGF-β in the lung, and latent TGF-β in the plasma, due to potent inflammatory and immune reactions with dysregulation of coagulations [132,133]. In severe COVID-19 with massive neutrophil infiltration in the lung, neutrophil elastase induces the release of TGF-β, which also recruits neutrophils into the lungs to create a positive-feedback loop in the activation and release of TGF-β [132]. Significantly, SARS-CoV-2 infection-induced lung epithelial cell apoptosis triggers the release of latent TGF-β, which is further activated by ROS, elastase, plasmin, integrin, furin, and matrix metalloproteinase [131]. These changes together with high pro-inflammatory cytokines trigger pulmonary edema and the development of ALI/ARDS. Therefore, both PDGF and TGF-β through NRP-1 receptors could participate in the pathogenesis of COVID-19 and AIS.

HIF is a transcription factor released in response to hypoxia and promotes survival in hypoxic conditions by the upregulation of various genes for enzyme-like glycolysis enzymes; this produce ATP independent of oxygen status, and VEGF, which promotes angiogenesis [134,135]. HIF is inactivated by HIF prolyl-hydroxylase, so HIF prolyl-hydroxylase inhibitors like desidustat and vadadustat inhibit renal HIF-α2; this increases the release of erythropoietin and is used for the treatment of anemia. However, the induction of HIF in normoxic conditions may promote inflammatory changes through the induction of STAT3 and the release of pro-inflammatory cytokines [136]. In addition, the HIF-pathway activator could have neuroprotective effects in the management of spinal cord injury and stroke [137,138].

In SARS-CoV-2 infection, hypoxia due to cardio-respiratory insufficiency induces cell death and apoptosis, releasing damage-associated molecule patterns (DAMPs), which activate the transcription of HIF-α1. The hypoxia also stabilizes HIF-α1 by the inhibition of prolyl hydroxylase mRNA through the activation of endothelial TLR4 [20]. Moreover, HIF-α1 reduces the entry of SARS-CoV-2 through the inhibition of ACE2, and transmembrane protein serine 2 (TMPPSS2) through the activation of ADAM17 [20]. However, prolonged HIF-α1 effect may lead to the development of ARDS due to the augmented release of pro-inflammatory cytokines and glycolysis-dependent generation of ROS [139].

In AIS, HIF-α1 is upregulated and activates microglia through CD36 to produce pro-inflammatory cytokines and ROS that interfere with the neurogenesis in the acute phase of AIS [140]. An experimental study by Wang et al. showed that hyperbaric oxygen therapy improves cerebral ischemia: reperfusion injury through the inhibition of autophagy and expression of HIF-α1 [141]. Similarly, HIF-α1 in AIS triggers pyroptotic and apoptotic neuronal cell deaths by activating the NLRP3 inflammasome [24,142]. In the contrast, the stabilization of HIF-α1 by the inhibition of prolyl hydroxylase could enhance neuroprotection via the regulation of neuronal Na-Ca exchanger [143]. An experimental study by Hamrick et al. demonstrated that the stabilization of HIF-α1 by small molecule iron chelator prevents oxidative stress-induced neuronal injury during AIS, and reduces infarct size by about 67% [144].

These findings indicate that high circulating HIF-α1 has a modulatory impact on the neurogenesis in AIS. Thus, high HIF-α1 in patients with COVID-19 due to cardio-respiratory-induced hypoxia may have detrimental or beneficial effects on the development of AIS and other neuronal complications.

**Figure 13:**
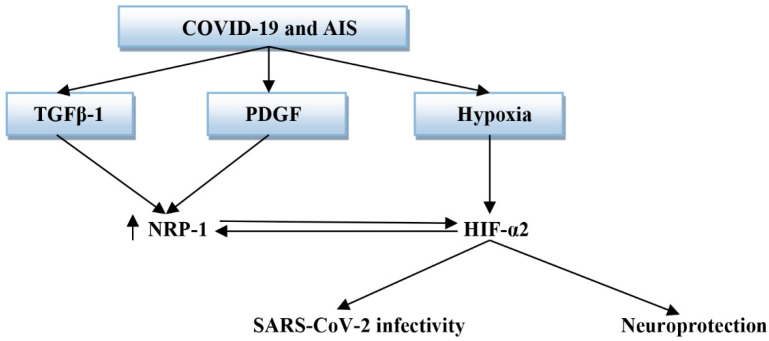
The potential role of the hypoxia-inducing factor (HIF), platelet-derived growth factor (PDGF), and transforming growth factor beta (TGF-β) in COVID-19 and acute ischemic stroke (AIS): PDGF and TGF-β activate the expression of neuropilin-1 (NRP-1), while hypoxia induces the expression of HIF, which has neuroprotective effect and reduces SARS-CoV-2 infectivity.

Furthermore, it has been confirmed that prolonged hypoxia upregulates the expression of NRP-1 through the expression of HIF-α1 [145]. Therefore, high NRP-1 in COVID-19 might be due to over-expressed HIF-α1. The net effects of PDGF, TGF-β, and HIF-α2 in COVID-19 patients with AIS are mainly mediated by the expression of NRP-1 [144,146,147] (Figure 13).

**Figure 14:**
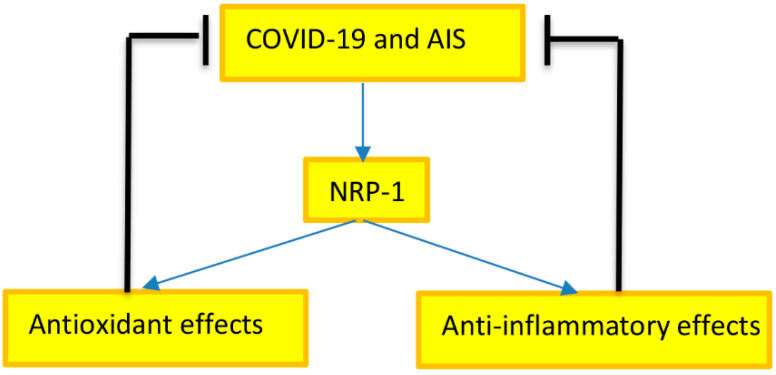
The potential role of neuropilin-1 (NRP-1) in COVID-19 and acute ischemic stroke (AIS).

The net effect of NRP-1 in COVID-19 with AIS is through the induction of anti-inflammatory and antioxidant effects which reduce the severity of both COVID-19 with AIS [Figure 14].

## 6. Conclusions

NRP-1 has an important role in the pathogenesis of COVID-19 and AIS, and could be the potential biomarker linking the development of AIS in COVID-19. Most studies suggest that high NRP-1 serum is linked with COVID-19 and AIS severities, but have not explained the underlying molecular mechanisms. However, the present study revealed that high NRP-1 serum in patients with COVID-19 and AIS depending on the previous findings might be a compensatory mechanism to overcome exaggerated immunity and neuroinflammation. Furthermore, it has been confirmed that prolonged hypoxia upregulates the expression of NRP-1 through the expression of HIF-α1. Therefore, high NRP-1 in COVID-19 might be due to over-expressed HIF-α1. These findings suggest that high circulating HIF-α1 has a modulatory impact on neurogenesis in AIS. Thus, high HIF-α1 in patients with COVID-19 due to cardio-respiratory-induced hypoxia may have detrimental or beneficial effects on the development of AIS and other neuronal complications. In addition, ANGPTL, a biomarker of ED, is elevated in COVID-19 due to the development of ED and associated thrombo-inflammation, which may cause the development of AIS. Thus, ANGPTL—mainly ANGPTL4 through the NRP-1 receptor—could be the potential link between COVID-19 and AIS. Moreover, semaP3A/NRP-1 pathway is required for TLR4 activation, which plays an integral role in the development of immunological disturbances during COVID-19 and AIS. Thus, the semaP3A/NRP-1 pathway is involved in the progression of harmful consequences in both AIS and COVID-19. The present review highlighted a potential link between COVID-19 and AIS mainly through the NRP-1 receptor. The present review cannot provide a final conclusion, and therefore in silico, experimental, in vitro, in vivo, preclinical, and clinical studies are recommended to confirm the potential role of NRP-1 in COVID-19, and to elucidate the pharmacological role of NRP-1 receptor agonists and antagonists in COVID-19.

## Data Availability

Not applicable.
